# The Surco canal, an ancient irrigation canal in Lima, Peru, and a citizens’ campaign for its protection

**DOI:** 10.1007/s12685-020-00257-1

**Published:** 2020-11-17

**Authors:** Javier Lizarzaburu

**Affiliations:** Vrije University, Amsterdam, The Netherlands

**Keywords:** Pre-Hispanic canals, Lima, Cultural heritage, Peruvian archaeology, Citizen campaign, Narratives

## Abstract

The present text discusses a number of pre-Hispanic irrigation canals in Lima, the Peruvian capital, often considered as the second largest city built on a desert. Some of the original canals are still in use but given that most have been excluded from official urban narratives, citizens are not aware of them and the role they still play in the city. A brief description of Lima’s canals through the ages is provided, including present day management and the risks affecting them. This paper focuses on the Surco canal and a citizens’ campaign carried out between 2016 and 2018 to raise awareness about it. The impact the campaign has had on the wider city is discussed. A general overview of the cultural context in which pre-Hispanic issues have historically been considered in Lima and how this campaign also tried to address them is included as well. As this paper is part of a special issue, Water History in the time of COVID-19, it has undergone modified peer review.

## Introduction

Like much of the pre-Hispanic past of Lima, a city that for centuries has focused primarily on its European legacy, the history of its canals has been largely omitted from official narratives. This was an issue often debated within the domain of archaeologists, architects and other specialists for decades, but outside academia few people knew about the canals and their role in the city. This lack of awareness resulted in the of people Lima considering this man-made infrastructure to be rivers. When the author started researching the subject, it became clear that their meaning went beyond the cultural and the archaeological: it was about a resource that was scarce in a city where it never rains. The symbolic meaning of the canals was high, as contemporary Lima seemed to ignore sustainable water use and had become a city with one of the highest levels of water wastage in the world.

The Peruvian capital Lima is a city of about eleven million people, or a third of the country’s total population, built on cliffs overlooking the ocean. Because of a phenomenon called the Humboldt Current, this section of tropical South America that would have been otherwise lush, rainy and warm, is in fact a desert territory (albeit with high humidity levels), like much of the Peruvian coast. The Humboldt Current runs counterclockwise in the Pacific Ocean, reaching the north of Chile where it turns north and runs along much of the Peruvian coast, before turning westwards again. This situation not only means that the coastal ocean is cold and rich in plankton, but also impedes the formation of rain and thus transforms the coast into a desert, which results, according to official data, into an annual average rainfall in Lima of 7 mm, which puts the city in the category of a ‘hyper arid’ desert (INEI 2017).

The Lima territory is crossed by three rivers that originate in the Andean glaciers, most of them at an altitude over 5000 m a.s.l. and the biggest of the three is the Rimac, which supplies water to Lima. Compared to the Nile, which runs through another desert-like city, Cairo, with a mean annual discharge of 2830 m^3^/s, the Rimac’s discharge is on average 25.8 m^3^/s. (Comisión de Regantes Surco-Huatica 2016). An additional factor that has an impact on the sustainability of Lima in the future is that over 50% of the Andean glaciers have melted in the last 50 years. Together with an intense, unplanned process of urbanization, this has made Lima one of the most water-stressed cities on the planet—a place vulnerable to climate change. More pertinently, the crisis of Covid-19 during 2020 has served to underline the observation that over one million people in the city have no access to running water.

In order to raise awareness, a campaign was launched in 2016 under the title ‘Canals of Lima: 2000 years watering life’ (Canales de Lima: 2000 años regando vida). Out of the original four main canals that existed in the lower valley of the Rimac River in Lima at the time of the arrival of the Europeans in the sixteenth century, the campaign focused on one of them: the Surco canal, the largest of them. Together with another canal in the area (the Huatica), water is supplied to an area of 1150 hectares, irrigating 711 parks in 17 (out of 43) districts of Lima where over three million people live. This represents 85% of all green areas in those parts of the city. The Surco canal along 29.5 km from its source in the Rimac River to the Pacific Ocean, and the Huatica, 15 km (Lizarzaburu 2018). Given the growing risks that affect them in city where planning is largely unregulated, the campaign stressed their importance for the future of Lima.

## Lima’s canals

It is intriguing that the canals survived in a city that has grown ten-fold in the last 50 years, destroying layers of its history. Perhaps more to the point, it seemed quite surprising that in such a pressurized urban context, these canals were still working for the city 2000 years after they were constructed. In the words of Peruvian architect Juan Gunther, a specialist in pre-Hispanic Lima, this ancient irrigation system was one of the most important works of hydraulic engineering in ancient Peru (Gunther 2012).

According to experts, among them archaeologist Sofia Chacaltana, there is a distinction to be made between the technology that allows for the digging of a single canal (which usually covers a very limited area of land), and the development of an irrigation system, consisting of a network of canals covering a larger territory, like the one that was established in Lima some 2000 years ago (Chacaltana 2016). The canals referred to in this article, like all the other ones that existed in the lower Rimac Valley, were formed by redirecting small sections of the Rimac River.

Metropolitan Lima starts at an altitude of 850 m a.s.l. (district of Chosica), as it is part of the Andean foothills which descend towards the Pacific Ocean—approximately 50 km away. Research carried out by the Riva Agüero Institute in Lima established that the section where the two canals referred to in this paper start irrigating the lower valley, begins at an altitude of 174 m (corresponding largely to where the historic city center stands today) and continues down to the ocean following a progressive gradient (Cogorno 2015). This slope towards the sea was perhaps the most important natural tool that allowed ancient groups to develop irrigation systems.

Originally, when they were used for agriculture, the main canals were six meters wide and 1.5 m deep, having an additional margin of another six meters on either side. Italian architect Adine Gavazzi, who has studied the Lima area, underlines that when the Europeans arrived in the sixteenth century, they did not understand the spatial layout of the territory, particularly, in the sense that it was a man-made landscape and not a natural one. For them, “the region went unnoticed in its territorial organization of cultivated fields, irrigation systems and roads” (Gavazzi 2014).

This process of transformation of the territory from a desert into productive valleys, through the digging of canals which took over two millennia, also happened in many other parts of the Peruvian coast. “This particularity differentiates us from the Egyptian, Indian and Chinese cultures, where development occurred along a single river, and it resembles the Sumerian and Mexican where development occurred thanks to the dominance of more than one river” (Gunther 2012).

## A brief history of Lima’s canals

American archaeologist Tom Dillehay and others have identified what is probably the oldest canal in Peru, located in the area of Nanchoc, in the Zaña Valley, in the region of Cajamarca (Northern Andes). It has been radiocarbon dated to 3400 BC (Dillehay et al. 2005). In the case of Lima, a study by the National Water Authority (ANA) suggested that the first canal may date to 2000 BC, being associated with the ancient temple of “Las Salinas”, in the district of El Agustino, next to the Rimac River (Casareto and Perez 2016).

During the period known as the Early Intermediate (200 BC–800 AD), the civilizations in this part of Peru created urban development that was different to earlier times. They showed a greater degree of agricultural management, thanks to large irrigation systems on both banks of the Rimac River (Canziani 2009). According to Narváez (2013), an archaeologist who is one of the main experts on Lima’s canals, “The complexity of the societies that existed during Pre-colonial times in this valley is expressed in the existence of monumental architecture, with huge pyramids, (…) forming complex and large settlements, in the existence of irrigation systems that covered several kilometers augmenting the available agricultural fields in the valley, and by the existence of a hierarchical system in the settlement pattern and in the exercise of power.”

Santiago Agurto, a Peruvian architect who worked on this period, believed that the construction of the early canal system “… allowed the people of the Lima culture to make their urban development independent from the river bank and be able to build ceremonial and administrative settlements in the heart of the valley, in wide and flat areas suitable to allow great urban development” (Agurto 1984). At least five different pre-Hispanic cultures have in turn settled in what is today the city of Lima, leaving behind a legacy of over 500 archaeological sites. Each of those cultures improved and/or expanded on what previously existed. The last ones to occupy these lands, before the Spanish, were the Incas, from c.1450 to c.1530.

Pre-Hispanic cultures were not only able to build extensively in the valley, but also to devote themselves to agriculture—so much so, that until the arrival of the Spanish, valley-based communities had managed to create a network of 250 km of canals, irrigating 30,000 hectares of land which had previously been desert (Gunther 2012). It was the extent of the irrigation system that caught the attention of the Spanish chroniclers in the sixteenth century, making reference to them as works that had been continually maintained over the ages in order to ensure water distribution throughout the valley (Flores Zúñiga 2009). “They took advantage of the water of the rivers, irrigating all the lands where it reached, and this work of their ditches was one of the greatest and most admirable they had, because they were well done and with so much order, that it is amazing to consider how, lacking our tools, they could dig and build them…” (Cobo 1882).

**Map 1:**
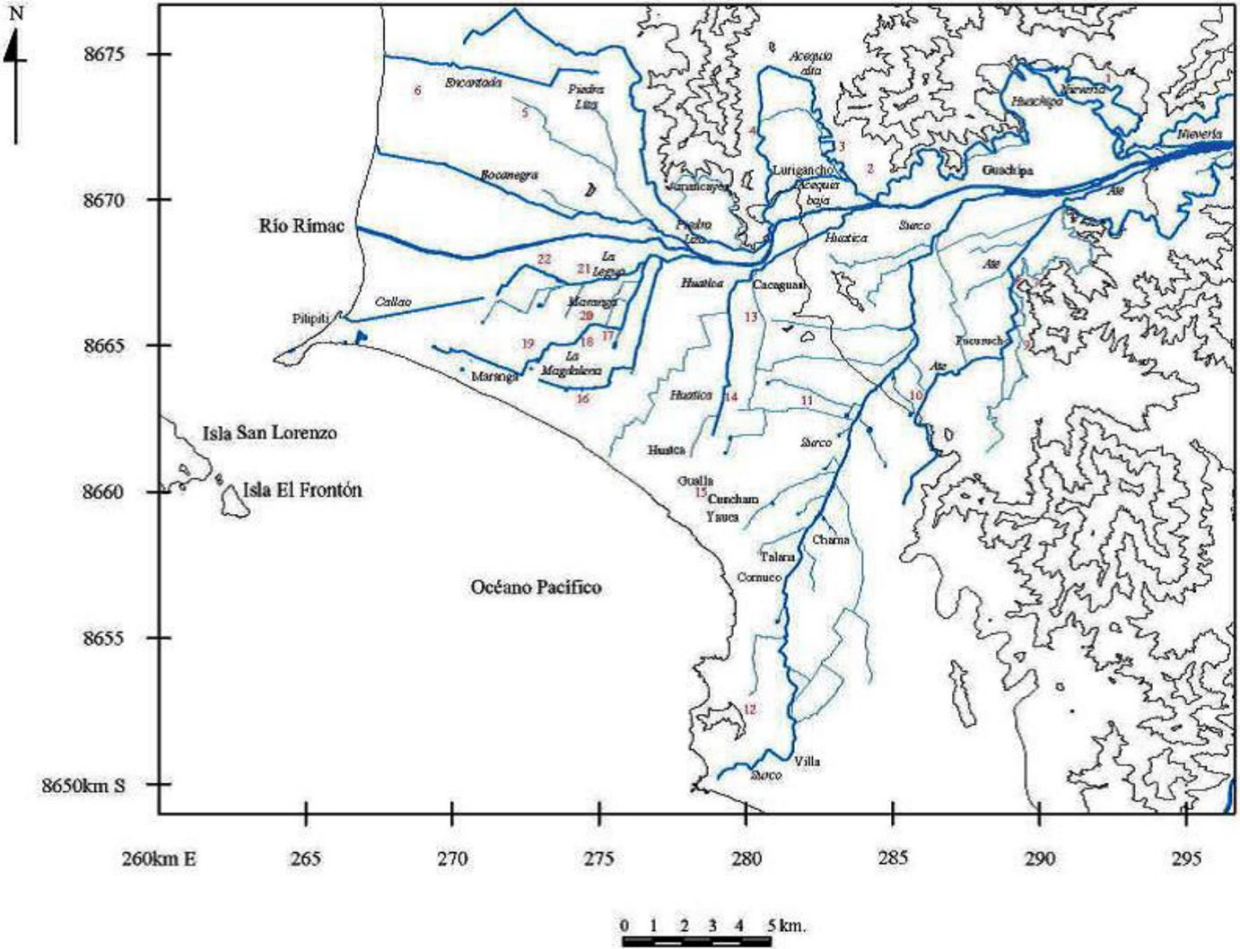
Lower Rimac River Valley and its network of canals, as it was at the beginning of the XX century. Image: Joaquín Narvaez

Agriculture continued to flourish with the new crops introduced by the Europeans, like olives, apples and sugar cane. Several records mention that until the mid-twentieth century, there were some 800 ‘haciendas’, estates and farms in Lima, all of them benefitting from the existing pre-Hispanic irrigation system. Peruvian historian Jorge Lossio, noted that even in the 1950s, Lima was still a self-sustaining city (Lossio 2003) (Map 1). The canals were used well into the mid-twentieth century, when the city entered into an accelerated and disorderly process of urban growth, which anthropologist José Matos Mar famously described as the “popular overflow” (Matos 1984). The canals were needed to water fewer and fewer areas, and partly for this reason many were narrowed down from the previous six meters to a width of one and a half meters. By the 1970s, most if not all of the former plantations had disappeared under the new city.

A new role was found for the surviving canals, as they were used for the irrigation of parks, gardens and green areas. At the same time, however, the vulnerability of these canals became more evident. Some of them, in other parts of the city, were destroyed or disappeared under the cement. Along the route of the Surco canal, there are still risk factors that remain a concern, such as unregulated urban growth, the invasion of private and/or public land, the dumping of waste, the illegal use of the water course, and route diversion without authorization, and these were some of the reasons why the campaign was launched (Lizarzaburu 2018).

## Canal management: the Irrigation Commission

It is thought that during pre-Hispanic times, control over the water distribution provided political leverage allowing different groups to live in relative peace. Historian María Rostowrowski noted that from the tenth to fifteenth centuries “[…] the lower valleys of Rimac and Lurin were integrated into a lordship named Ichma that encompassed smaller lordships, each divided into halves with land around the main irrigation canals […]” (Rostowrowski 1978). During the Spanish colonial period, the institution of Water Judges was created in order to deal with the multiple legal disputes that arose when the new owners of the land settled and did not understand the workings or the philosophy behind the traditional management of these waterways.

Dominguez (1988) mentions that the treatise devised by Cerdán y Pontero in 1793 was in force in Peru until 1911, when a new “Water Code” was announced, creating the Water and Agriculture Directorate. A few years later, the Irrigation Commissions were set up, which today are part of the National Water Authority, ANA, a branch of the Ministry of Agriculture. Currently, the Rimac River Basin Users Board covers a much bigger area than the one described in this article. It includes 17 Irrigation Commissions, in charge of the irrigation of 4700 hectares, mostly agricultural land outside metropolitan Lima. The Surco-Huatica commission is one of these 17 organizations. According to the information provided by the Surco-Huatica Irrigation Commission, the current system consists of a main (or central) canal, also called the mother-canal, with other canals originating from the main one (primary, secondary and tertiary canals, as well as ditches, depending on their width and length) (Lizarzaburu 2018).

**Map 2:**
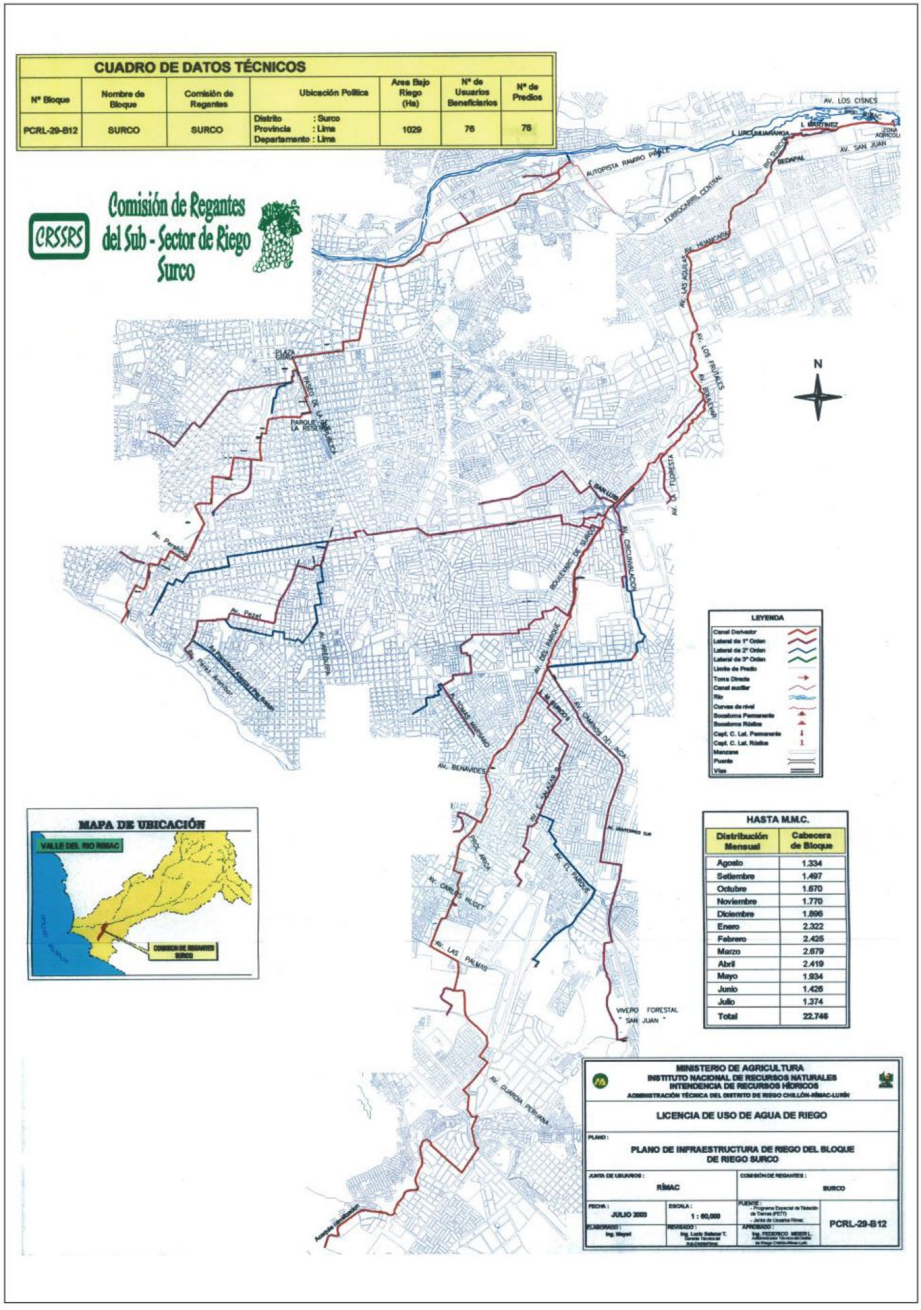
Current route of the Surco and Huatica canals. *Sourc*e Irrigation Commission

From the total area within the Users Board, 1150 hectares are irrigated by the Surco and Huatica canals, under the management of the Irrigation Commission (Map 2). This is the equivalent of 3.1 square meters of green areas per citizen, or one third of the 9 square meters recommended by the World Health Organization (WHO) as a healthy minimum. The Irrigation Commission is responsible for the central or mother-canal, while the minor canals are under the authority of each municipality. Among its members are local authorities (16 municipal districts), 10 public institutions (including the city’s main two cemeteries), 7 private institutions (including three universities and four clubs—the main golf club in Lima being one of them), as well as a small number of urban farmers (Table 1). Together, the 69 users each pay a rate of 0.10 Peruvian cents per cubic meter of water (US$ 0.028 approx.).

**Table 1:** Detail of the green areas irrigated by the Surco canal in 13 municipal districts of Lima

Users	Hectares	Parks
Municipalities/districts:		
Ate	7	55
La Molina	3	14
La Victoria	50	65
Lima Metropolitana	10	5
Lince	9.7	9
Miraflores	120	58
San Borja	149	83
San Isidro	187.5	48
San Luis	13	27
Santa Anita	20	44
Surco	157.5	226
Surquillo	14	43
Chorrillos (unofficial)	(Undetermined)	677
Universities:		
UNIFE	3	
Ricardo Palma	8	
San Martín	2	
Clubs:		
Jockey Club del Perú	54	
Lima Golf San Isidro	45	

*Source* Irrigation Commission Surco-Huatica

Recently, tensions have arisen due to changes introduced by the Ministry of Agriculture. Until 2018, the Irrigation Commissions were the sole operators of the canal infrastructure. Now, with the new Operators Regulation, Irrigation Boards have become the operators and the Irrigation Commissions have become the administrators. This means that the operator is in charge of the collection of tariffs and the maintenance of the infrastructure, which was previously the responsibility of the Commissions. In addition, if the Commission wants to undertake work on the canal, it must request authorization from the Board. To date, these changes have not been implemented and the system continues to operate under the old model.

## The campaign

Aside from the risk factors affecting them, when research began on the canals, some striking facts immediately came to light related to water availability and water consumption in Lima:

- Even though Peru ranks number 8 in potential water availability in the world, with 1.89% of the total (FAO 2013), 98% of those resources go directly into the Atlantic basin.
- The majority of the population, 66%, live close to the Pacific basin, where water availability is calculated at 2.2% (ANA).
- The WHO recommends an average water usage of 100 lt. per person per day. The average in Lima is 250 lt. pp. pd. (Ministry of Housing 2018).
- As usual, averages hide substantial inequalities. The richest districts consume the most in Lima: San Isidro, 477 lt. pp. pd., and Miraflores, 436 lt. pp. pd.
- The districts with the lowest levels of water consumption are Cieneguilla, 44 lt. pp. pd. and one of the poorest, Lurigancho-Chosica, with 26 lt. pp. pd.
- Water is available in the poorest areas for only a few hours each day.
- In comparison, in the city of Amsterdam, the average is 133 lt. pp. pd. (Waternet), and Paris 143 lt. pp. pd. down from 151 in 2008 (Statista).

If people are using so much water in a place where there is so little, it seems evident that there is a lack of awareness of the current situation. Even though the campaign considered in this text was not directly aimed at raising awareness of domestic water wastage, it is nevertheless related, since the water the canals use originates from the same source as the drinking water that feeds the needs of the city of Lima. This is dealt with in the last section.

The campaign was officially approved during an Extraordinary General Assembly of the Irrigation Commission, on February 29, 2016. The broader scenario was to secure recognition of segments of the Surco Canal as National Cultural Heritage, to be granted by the Ministry of Culture. In addition to the main objective others were identified including raising awareness of the scarcity of water as a natural resource, informing citizens of the environmental importance of the canals, and letting people know that the existing infrastructure—instead of rivers—were a man-made network of pre-Hispanic origin. All these goals needed to be framed in a context of cultural, historical and environmental value.

In order to get academic and professional support, an Advisory Council was also created, which included architects, experts on the pre-Hispanic period, archaeologists, and other professionals keen on offering their support. (Here onwards, where actions included the author, members of the Commission and the Advisory Council, they are referred to as ‘the Group’). With the Surco being a working canal, it would have been impossible to ask for the declaration of National Heritage for the whole length of it. Instead, one of the archaeologists in the Advisory Council, Joaquín Narváez, undertook an additional study and wrote a report identifying four sections of it, that would have remained unchanged since pre-colonial times, and these were put forward as possible segments for declaration. As part of this process with the Ministry of Culture, a supporting documentation was required that documented and explained why the Group thought this declaration was necessary. Much of the information in this essay comes from that original document and later from a book published on the subject (see Useful Links).

For Castells (2009), people understand and communicate better through metaphors, because this allows messages to engage with the reader more effectively. This approach was employed when writing articles on the canals for the Lima Milenaria blog. Like almost everything pre-Hispanic in Lima, the canals had survived at the margins of official narratives, so it was important to put them in a context of practical benefit for the citizens as well as to attempt to generate links between the present day readers and the ancient people of Lima who were, after all, the ones who had given them this great inheritance. This emphasis was also conveyed to other media when they wrote on the subject.

In total, 20 stories were written in the blog; nearly 100 stories related to the canals and the campaign were published in the national and international media, as well as local TV and radio. The social media campaign reached over two million people. Over 25 public meetings were held in citizens’ associations, municipal auditoriums and cultural centers, explaining the origin of the canals and the objectives of the campaign. As the campaign expanded, well-respected artists and influencers offered their support, some of them in the form of video clips. In order to bring the campaign to a close, a large public event was held in one of the parks where a section of the open canal remains. The activities incorporated an ancestral ritual celebrating the pre-Hispanic infrastructure, a walk along the route of the Huatica canal, a bicycle ride along the route of the Surco canal, and heritage workshops for children, all organized by different groups of citizens who were also supporting the campaign.

## By way of conclusion

The application was submitted to the ministry in September 2016 and the process took longer than expected. This was mainly due to the fact that until then, the officials had never had to consider declaring a pre-Hispanic canal as National Cultural Heritage. The examples for doing so were in general limited to monumental architecture and monumental engineering works, as there was no precedent for something as seemingly modest (yet so crucial for the city) as these canals. However, the two years it took to complete the process allowed for a number of follow-up meetings, where members of the Group engaged in working meetings with experts at the ministry. The Ministry of Culture also carried out its own research into the canals. In March 2019, Vice-Ministerial Resolution No. 041-2019-VMPCIC-MC was officially published, in which one of the four segments suggested by the Group was declared to be part of Peru’s National Cultural Heritage (El Peruano 2019). According to informal communications with the author in 2019, the process for the declaration of the other segments has been completed, pending of clearance of legal property rules.

It has not been possible to measure the precise level of awareness raised by the campaign during those two years, but there were several positive signs that gave an idea that some impact had been achieved. One of them, at an academic level, was an event titled Limapolis 2017. In March 2017, an international workshop was organized by the Faculty of Architecture and Urbanism at the Pontifical Catholic University of Peru, PUCP, to study the Surco canal. It was an event dedicated to urban issues that focused on the possibilities of urban regeneration, environmental improvement, enhancement of heritage, and promotion of social inclusion from the route of the Surco Canal through the city. In total, 16 architects, both from Peru and abroad, spent one week developing initial strategies for the integration of the 30 km of this canal into the city as a public space (Benavides 2018).

Later, as a consequence of the health emergency generated by the Coronavirus in 2020, the Peruvian government launched a series of economic reactivation measures, especially investment in infrastructure. In this context, the Ministry of Agriculture has promised aid of up to 150 million Peruvian soles (over US$ 40 million), and in May 2020 approved an initial list of works for 91 million Peruvian soles (over US$25 million), which includes 4126 maintenance projects for irrigation canals in 22 departments in the country. The current president of the Surco-Huatica Irrigation Commission, Mr. Mario Ichiki, informed the author via e-mail that the Surco and Huatica canals will be one of the beneficiaries of this fund, with a different but also very significant new approach to its management.

This will be based on replacing the use of river water as its main source and moving towards the use of treated wastewater, thus improving the efficiency of the irrigation system in a growing scenario of water scarcity. According to existing data, only 0.09% of public parks and gardens are irrigated with residual treated water. On the other hand, La Chira and Taboada treatment plants discharge 550 million m^3^ of processed water into the sea.

Additionally, the Commission is working with the Ministry of the Environment to produce a Master Plan for the recuperation of the Surco and Huatica canals. The draft document sent to the author includes, for the first time in ministerial documents, subjects like the preservation of the cultural heritage associated with the canals and the generation of identity and links between citizens and their cultural heritage.

Meanwhile, in another communication with the author via e-mail, the Director of the Office for the Historic Center of Lima, PROLIMA, Mr. Luis Martín Bogdanovich, mentioned, as a way of evaluating the campaign, that it had been decisive in the creation of a narrative based on the value of a heritage previously invisible for decades. The progress made has served to incorporate the protection and recuperation of the extant canals within the Historic Center in the Master Plan, recently approved by Ordinance 2194–2019 MML.

Mr Bogdanovich added that “to date, PROLIMA has been working on the preliminary plans for two public spaces where we could show the canals: the Monserrate Square, associated with the Maranga canal; and the Carrion Square, associated with the Matute canal, a primary canal diverted from the Huatica. Likewise, from the recently created archaeology division at PROLIMA, an archaeological research project has been completed to explore the remains of the canals that passed through the Historic Center, which will also be submitted to the Ministry of Culture.” So it is possible to say that there is hope for the ancient canals of Lima.

## Data Availability

All data and materials comply with field standards.
